# P-554. Anxiety and Depression are Associated with Poor Antiretroviral Adherence in People Living with HIV: A Real-World Survey in Europe

**DOI:** 10.1093/ofid/ofae631.753

**Published:** 2025-01-29

**Authors:** Fritha Hennessy, Will Ambler, Tim Holbrook

**Affiliations:** Adelphi Real World, Bollington, United Kingdom, Bollington, England, United Kingdom; Adelphi Real World, Bollington, England, United Kingdom; Adelphi Real World, Bollington, United Kingdom, Bollington, England, United Kingdom

## Abstract

**Background:**

High adherence to antiretrovirals (ARV) is vital for people living with HIV (PWH) to ensure undetectable viral load and prevent viral transmission. Various factors can affect adherence, including mental health, although little is known about the impact of anxiety and depression in Europe. Therefore, we aimed to assess adherence to ARV based on PWH’s self-reported level of anxiety or depression.

**Figure 1.**
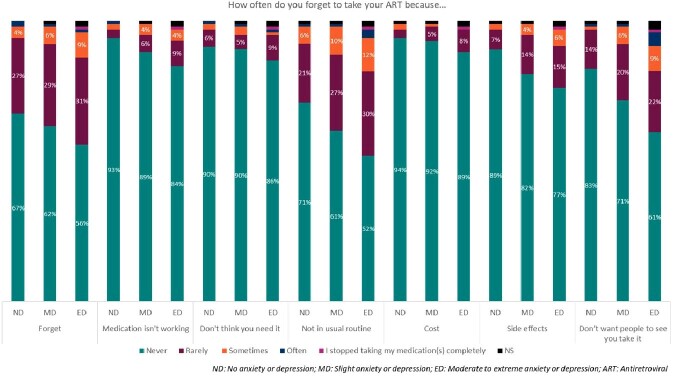
PWH response to “How often do you forget to take your ARV because…”

**Methods:**

Data were drawn from the Adelphi HIV Disease Specific Programme™, a real-world, cross-sectional survey with retrospective medical chart data collection of PWH in Europe (France, Germany, Italy, Spain, United Kingdom) between November 2022 and June 2023. PWH self-completed surveys, reporting ARV adherence via the Adelphi Adherence Questionnaire (ADAQ), a 0 to 4 scale, with lower scores indicating greater adherence. Analyses were descriptive.

**Figure:**
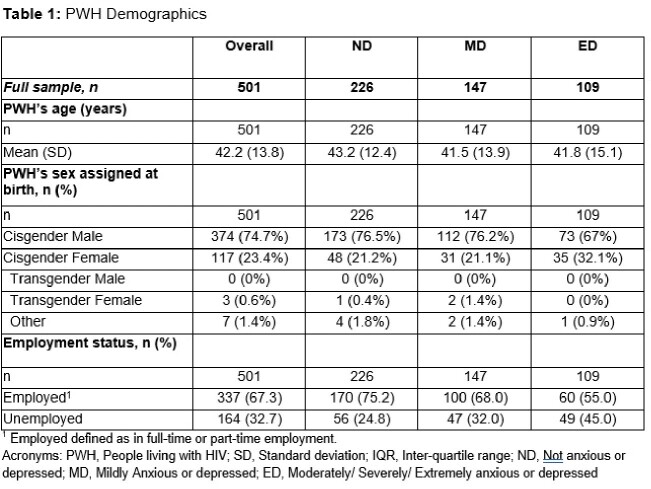

**Results:**

Of the 501 PWH included, mean (standard deviation, SD) age was 42.2 (13.8) years and 74% were cisgender male (Table 1). PWH (n=482) self-reported being not (ND, n=226), mildly (MD, n=147), or moderately/severely/extremely (ED, n=109) anxious or depressed. PWH with ED had a notably low mean CD4+ count (Table 2). Levels of viral suppression ranged from 75.5% of MD PWH to 54.2% of ED. Worrying about forgetting to take HIV medication was reported by many ED and MD PWH. These PWH also reported that taking their ARV was an uncomfortable reminder of their HIV status (Table 3).

PWH with MD and ED had a mean (SD) ADAQ score of 0.28 (0.37) and 0.38 (0.50), respectively, compared to 0.20 [0.31] for ND. ED PWH reported lack of adherence due to being in a different routine (48%), of not wanting others to see them taking it (39%), or side effects (23%), this was 39%, 29%, 18% for ND PWH (Figure 1).

**Figure:**
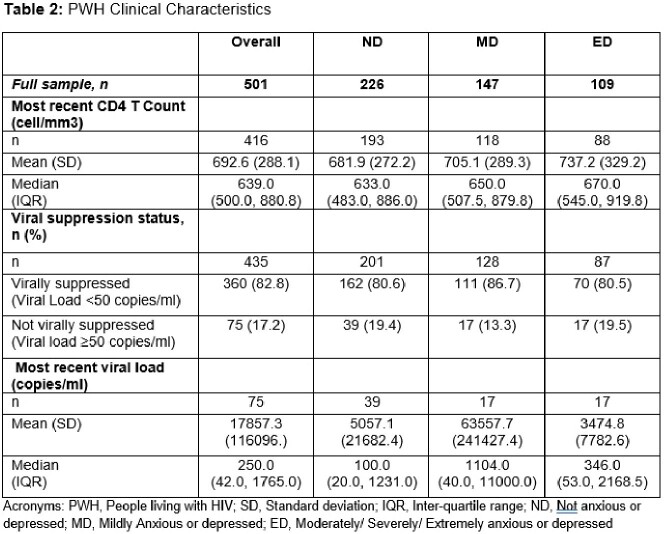

**Conclusion:**

Anxiety and depression impact PWH’s adherence to their treatment. Higher levels of anxiety and depression are associated with low treatment adherence in PWH. Suitable mental health support, or other targeted support for PWH with anxiety or depression, may improve adherence and outcomes.

**Figure:**
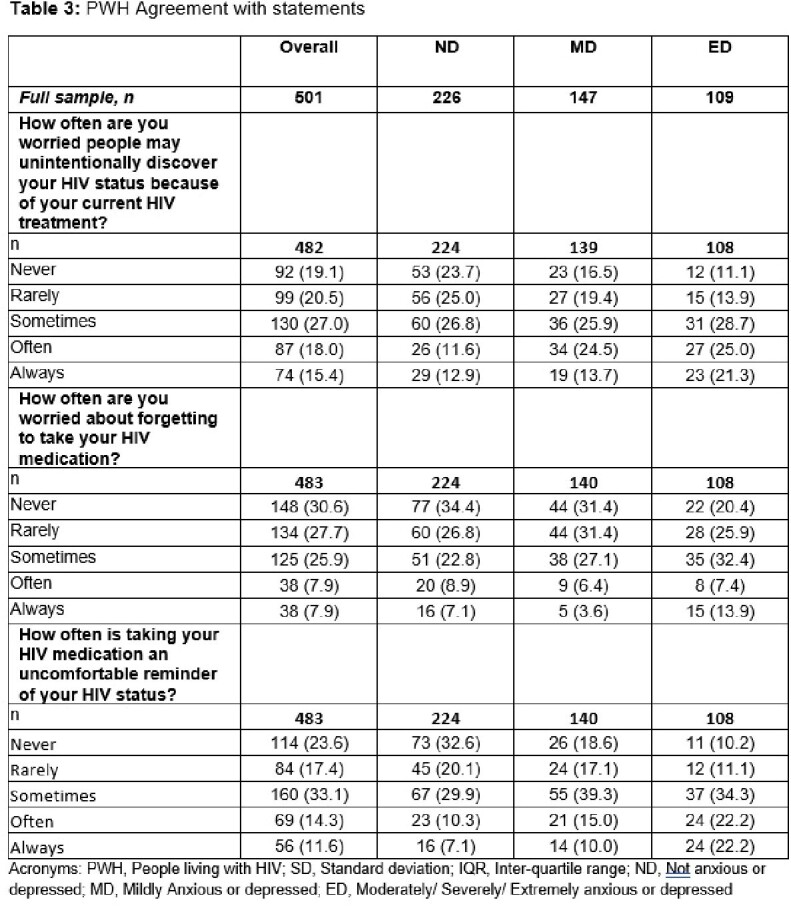

**Disclosures:**

**Fritha Hennessy, PhD**, Adelphi Real World: Employee|ViiV Healthcare: The Adelphi Real World Disease Specific Programme is wholly owned by Adelphi Real World; ViiV healthcare is one subscriber and paid for the analysis **Will Ambler, PhD**, Adelphi Real World: Employee|ViiV Healthcare: The Adelphi Real World Disease Specific Programme is wholly owned by Adelphi Real World; ViiV healthcare is one subscriber and paid for the analysis **Tim Holbrook, BSc**, Adelphi Real World: Employee|ViiV Healthcare: The Adelphi Real World Disease Specific Programme is wholly owned by Adelphi Real World; ViiV healthcare is one subscriber and paid for the analysis

